# Treatment of nonarteritic anterior ischemic optic neuropathy with an endothelin antagonist: ENDOTHELION (*ENDOTHEL*in antagonist receptor in *I*schemic *O*ptic *N*europathy)—a multicentre randomised controlled trial protocol

**DOI:** 10.1186/s13063-022-06786-9

**Published:** 2022-10-29

**Authors:** Christophe Chiquet, Catherine Vignal, Philippe Gohier, Emmanuel Heron, Gilles Thuret, Marie Bénédicte Rougier, Audrey Lehmann, Laurent Flet, Jean-Louis Quesada, Mathieu Roustit, Dan Milea, Jean-Louis Pepin, For the ENDOTHELION group

**Affiliations:** 1 Department of Ophthalmology, Grenoble Alpes University Hospital, 38043 Grenoble, France; 2 HP2 Laboratory, INSERM U1300, Grenoble Alpes University, Grenoble, France; 3 Department of Ophthalmology, Adolphe de Rothschild Foundation Hospital, 75940 Paris Cedex 19, France; 4 Department of Ophthalmology and Internal Medicine, Quinze-Vingts National Hospital Centre for Ophthalmology, 75940 Paris, France; 5 Department of Ophthalmology, CHU de Angers, 49100 Angers, France; 6 Department of Ophthalmology, CHU de Saint-Etienne, 42055 Saint-Etienne, France; 7 Department of Ophthalmology, Hôpital Pellegrin, CHU de Bordeaux, 33000 Bordeaux, France; 8 Department de Pharmacy, Grenoble-Alpes University Hospital, 38043 Grenoble, France; 9 Department of Pharmacy, PTMC –Clinical Trials, CHU de Nantes, 44000 Nantes, France; 10 Clinical Pharmacology Unit, Grenoble Alpes University Hospital, Grenoble, France; 11 Visual Neuroscience Group, Singapore Eye Research Institute (SERI), Singapore National Eye Institute (SNEC), Duke-NUS Graduate Medical School, Singapore, Singapore; 12 Pôle Thorax et Vaisseaux, Grenoble Alpes University Hospital, Grenoble, France

**Keywords:** Nonarteritic anterior ischaemic optic neuropathy (NAAION), Endothelin, Bosentan, Visual field, Randomised controlled trial

## Abstract

**Background:**

Nonarteritic anterior ischemic optic neuropathy (NAAION) is a major cause of blindness in individuals over 50 years of age, with no available effective treatment. The oral dual endothelin receptor antagonist, bosentan, increases retinal optic nerve head blood flow in healthy humans and glaucoma patients. The objective of this trial is to assess the efficacy of bosentan administered at the acute stage in improving outcomes in NAAION patients.

**Methods:**

ENDOTHELION (ENDOTHELin antagonist receptor in Ischemic Optic Neuropathy) is a phase III, interventional, prospective, multicentre, placebo-controlled randomised double-blind clinical trial. The primary outcome is change in the visual field mean deviation (MD) at 3 months (Humphrey 30-2 SITA standard programme). Secondary outcomes include MD and visual acuity changes up to 24 months, changes in peripapillary retinal nerve fibre and macular ganglion cell layer thickness in the affected eye, as measured by optical coherence tomography, rate of NAAION bilateralisation at 2 years, and quality-of-life. Patients over 50 years of age presenting with typical NAAION of recent onset (less than 21 days) are randomly assigned to either 125 mg oral bosentan or placebo, twice a day, during 8 weeks. Besides visits during the treatment phase, patients attend follow-up visits at 2, 3, 6, 12 and 24 months. The inclusion of patients began in August 2015 at five French University hospital ophthalmology departments and two specialised ophthalmology centres. It is planned to include 86 patients in this trial. To date we have included 72 patients and 49 have completed the full follow-up process.

**Discussion:**

An endothelin receptor antagonist is a potential approach to improving the anatomical and functional prognosis of patients with NAAION. This multicentre double-blind randomised controlled trial is an opportunity to assess (1) the effect of bosentan on the structure and function of the optic nerve in NAAION, at 3 months, (2) the effect of bosentan on the bilateralisation rate at 24 months and (3) the tolerance profile of bosentan in this population.

**Trial registration:**

ClinicalTrials.gov NCT02377271. Registered on March 3, 2015.

## Administrative information

Note: the numbers in curly brackets in this protocol refer to SPIRIT checklist item numbers. The order of the items has been modified to group similar items (see https://trialsjournal.biomedcentral.com/submission-guidelines/preparing-your-manuscript#preparing+main+manuscript+text.

**Table:** 

**Title {1}**	TREATMENT OF NONARTERITIC ANTERIOR ISCHEMIC OPTIC NEUROPATHY WITH AN ENDOTHELIN ANTAGONIST: ENDOTHELION, A MULTICENTRE RANDOMISED CONTROLLED TRIAL
**Trial registration {2a and 2b}.**	ClinicalTrials.gov Identifier: NCT02377271. Registered on March 3, 2015
**Protocol version {3}**	Version 8, 30 May 2020
**Funding {4}**	ARFO (Association for Research and Training in Ophthalmology), Grenoble, France; AgirADom, Grenoble, France; Foundation VISIO, France; PHRC (Hospital clinical research project), Ministry of Health, France; Grenoble Alpes University Hospital, France.
**Author details {5a}**	**Grenoble University Hospital**, France: Christophe CHIQUET, Jean-Louis PEPIN, Olivier ORMEZZANO, Matthieu ROUSTIT, Bertrand TOUSSAINT, Candice TROCME, Marie-Laure GAVARD, Jean-Louis QUESADA **Bordeaux University Hospital**, France: Marie-Bénédicte ROUGIER **Saint-Etienne University Hospital**, France: Gilles THURET **Angers University Hospital**, France: Philippe GOHIER, Dan MILEA **Adolphe de Rothschild Foundation Hospital**, Paris, France: Catherine VIGNAL **Quinze-Vingts National Hospital Center for Ophthalmology, Paris, France**: Catherine VIGNAL, Emmanuel HERON
**Name and contact information of the trial sponsor {5b}**	**University Hospital of Grenoble-Alpes, Department of clinical research (DRCI)** (Dr Camille DUCKI, cducki@chu-grenoble.fr)
**Role of sponsor {5c}**	Approval of study protocol, management of funding, and study monitoring

## Introduction

### Background and rationale {6a}

Nonarteritic anterior ischaemic optic neuropathy (NAAION) is the most frequent acute optic neuropathy after 50 years of age, with a general annual incidence worldwide of 10 per 100,000 individuals, and estimated at 6000 cases/year in France [1]. The pathophysiology of the disease is not well understood, being probably related to acute hypoperfusion in the short posterior ciliary arteries resulting in damage to the papillary microcirculation. This condition has been associated with various predisposing systemic factors [2] such as obstructive sleep apnoea syndrome (OSAS) in 75–80% cases [3–6], hypertension in 38–60% [7–10], diabetes in 12–23% [7, 8], dyslipidemia in 36–48% [10, 11] and anatomic ocular factors (small papillary excavation [12–15]).

The prognosis of NAAION is most often unfavourable, due to the irreversible loss of central visual acuity and visual field (VF). While some studies have reported slight spontaneous improvement (22%) [16], others reported no improvement or the deterioration of visual acuity (VA) or the VF [9, 17]. In eyes initially presenting minimum to slight VF defects, worsening was observed at 3 months after the first visit in 26% of cases and at 2 years in 27% of cases [16].

It is crucial to develop new therapeutic strategies for the acute stage of the disease so as to reduce optic nerve damage. Although corticosteroids and levodopa have been tried, these treatments have not been fully validated [18–23]. Other therapeutic strategies have been proposed, but their efficacy has not been demonstrated. These include posterior vitrectomy in patients with a small optic nerve head (ONH) and partial posterior detachment of the vitreous [24], radial neurotomy [25], intravitreal injection of triamcinolone [26, 27], electrical transcorneal stimulation [28], instillation of brimonidine [29] and LDL apheresis [30]. No validated treatment currently exists for this ischaemic optic neuropathy. Table 1 summarises the main recent studies on NAAION treatments.

**Table 1:** Summary of studies on the treatment of NAAION (2008–2020)

Author (year)	Grade of evidence	Number of patients	Type of study	Treatment window (range)	Treatment (dose)	Outcome	Confounding factors considered
Guerriero (2009) [30]	III	20 treated 10 untreated	Case–control	N/A	LDL apheresis	Short-term improvement (in the 3 months after onset of the disease) in the MD (−11.08 ± 6.51 vs −16.53 ± 10.03, *P* = 0.03; −17 ± 5.24 vs −14.14 ± 9.42), but no benefit at 6 months	N/A
Modarres (2011) [31]	IV	31	Case series	(Range 3–22 days)	Intravitreal erythropoietin (2000 units)	VA improved in 27 eyes (87%), 20 eyes (64.5%) showed ≥3 lines of visual improvement at 3 months (*P* < 0.001)	N/A
Rebolleda (2013) [23]	III	10 treated 27 untreated	Case–control	2 weeks	Oral prednisolone (80 mg daily, tapering dose)	No significant difference between the median change in VA (in the treated group median change in LogMAR VA between the initial and 6-month visit was −0.032 (±0.21) [−0.3; 0.04]), in MD (in treated group median change in MD was −0.56 dB (±5.03) [−3.6; 0.7] ), in PSD (in treated group median change in PSD was −0.02 (±1.9) [−1.6; 0.6]) and average loss in RNFL (in the treated group median change in average RNFLT was 150.5 (±64.9) [119.8; 212.7])	Hypertension, diabetes mellitus, hypercholesterolaemia, aspirin use
Saatci (2013) [32]	III	17 total (16 patients)	Retrospective	(2–15 days)	Intravitreal ranibizumab (0.5 mg)	VA improvement in 14/17 (BCVA 1.45 ± 0.88 vs 0.77 ± 0.70 LogMar at 1st year), disc swelling improvement in 17 (RNFL was 210 ± 38 microns vs 57 ± 18 microns at 1st year)	N/A
Radoi (2014) [33]	III	21 treated 15 untreated	Retrospective	1 month	Intravitreal triamcinolone (4 mg)	At 6 months, higher proportion of patients with improved VA of more than 1 line (> 5 letters) in the injected group [15 patients (71%)] vs the untreated group [2 patients (13%)] (*P* = 0.0009). Mean variation of VA letters at 1 month was 13.81 in treated group vs 0.33 in untreated group (*P* = 0.003) Mean variation of MD at 1 month was −1.33 (±1.9) in treated group vs 1.77 (±2.5) in untreated group (*P* = 0.007)	N/A
Rootman (2013) [34]	III	17 treated 8 untreated	Prospective, non-randomised controlled	15 days	Intravitreal bevacizumab (1.25 mg)	No VA or VF or disc swelling improvement (*P* = 0.3, *P* = 0.4 and *P* = 0.1 respectively)	Non-insulin-dependent diabetes, hypertension, smoking
Prokosch (2014) [35]	III	30 treated patients and 30 untreated patients	Prospective, randomised controlled	3 days	Pentoxifylline (PFX) IV vs Pentoxifylline + fluocortolone (FC)	Change BCVA for PFX + FC patients was 0.11 ± 0.14 after 3 days and 0.21 ± 0.19 after 6 months, while there was no change in the BCVA score in PFX group at either of these time points (mean change BCVA after 3 days 0.0 ± 0.18, after 6 days 0.05 ± 0.19; *P* < 0.002 and *P* < 0.001, respectively). At 6 months after starting therapy MD in PFX patients was −14.4 ± 9.2 and −16.74 ± 3 in PFX + FC patients, comparison of the data showed no significant difference (*P* < 0.2)	N/A
Kinori (2014) [36]	III	24 treated 24 untreated	Retrospective case–control	14 days	Methylprednisolone (1 g/day)	Mean initial VA was 20/70 (LogMAR 0.54 ± 0.67) in the treated group and 20/69 (LogMAR 0.54 ± 0.49) in the control group (*P* = 0.8). At the end of follow-up, VA acuity for control group was 20/80 (0.60 in LogMAR) in the treated group and 20/53 (0.42 in LogMAR) in the control group (*P* = 0.3). VF showed defects in 2.4 ± 0.8 in the treated group and 2.0 ± 0.6 quadrants in the control group (*P* = 0.007). At final visit, quadrant involvement was 2.6 ± 0.9 in treated group and 2.2 ± 0.7 in control group (*P* = 0.07) No statistical difference was found between groups at the end of follow-up (*P* = 0.2).	Diabetes mellitus, dyslipidaemia, hypertension, ischaemic heart disease and smoking
Zhu (2015) [37]	IV	16	Case series	(6–18 days)	Enhanced extracorporeal counterpulsation (EECP)	10 eyes (62.5%) showed improvement of 3 or more Snellen lines. The median LogMAR VA in NAAION eyes was 0.92 [0.32; 1.92] before EECP and was 0.40 [0.22; 0.90] after the last EECP treatment. Significant difference in median change in VA in NAAION eyes between before EECP and after 12 h EECP (*P* = 0.003). Median MD before EECP was −15.90 dB (SD 5.81) vs −15.14 dB (SD 5.02) after 12-h EECP (*P* = 0.049)	Diabetes mellitus, dyslipidaemia, hypertension, history of ischaemic heart disease, smoking, previous cerebrovascular accident, alcoholism
Lyttle (2016) [38]	III	33 treated 26 untreated	Retrospective	15 days	Levodopa (100 mg levodopa / 25 mg carbidopa three times daily)	Among patients with ≤ 20/60 initial VA, treated participants had significant improvement (*P* < 0.0001) in the mean change from initial to final LogMAR VA of −0.74 ± 0.56 (95% CI, −0.98 to −0.50), while the mean change for the control group of−0.37 ± 1.09 (95% CI estimate, −1.00 to +0.26) was not significant (*P* = 0.23). A significant difference between groups was observed (*P* = 0.0086) with19/23 (83%) in the treated group improving and none got worse, compared with 6/14 (43%) in the control group improving while four (29%) worsened. The treated group had worse mean initial VF MD at −18.9 dB (±8.8) compared to the mean initial visual field MD of controls of −13.3 dB (±8.5) (*P* = 0.04). The groups were not found to be significantly different (*P* = 0.23), with the estimated difference in means at follow-up being 1.78 dB (95% CI, −1.19 to +4.74). The treated group had a mean reduction of 57.1% RNFLT to 73.2 (±33.2) μm, and control eyes had a mean reduction of 62.5% RNFLT to 85.4 (±19.0) μm compared to the initial RNFL thickness, but this was not statistically significant. No significant difference on VF and disc swelling (*P* = 0.23 and *P* = 0.75, respectively)	N/A
Sanjari (2016) [39]	IV	13	Case series	14 days	Intravitreal Fasudil (0.025 mg/0.05 mL)	At M3, BCVA improved from 1.69 ± 0.55 LogMAR at baseline to 0.93 ± 0.51 LogMAR (*P* = 0.004), RNFLT decreased from 173.5 ± 29.28 μm to 62.9 ± 5.97 μm (*P* = 0.003) and MD values changed from 24.60 ± 3.80 to 20.5 ± 6.50 (*P* = 0.005)	N/A
Aftab (2016) [40]	IV	24	Case series	4 weeks	Heparin IV / Warfarin PO	Significant VA improvement in 16 (66.7%), worsening in 1 (4%), average improvement was 5.6 LogMAR lines	Hypertension, dyslipidaemia, diabetes mellitus
Pakravan (2016) [21]	III	90 total 30 untreated	Randomised controlled	N/A	Steroid / normobaric oxygen with mask	Mean initial BCVA was 1.02 ± 0.63, 1.05 ± 0.7 and 0.76 ± 0.5 LogMAR in groups 1 (control), 2 (steroid) and 3 (oxygen), respectively (*P* = 0.293); corresponding values were 0.8 ± 0.45, 0.84 ± 0.45 and 0.58 ± 0.4 at month 1 (*P* = 0.127, 0.19 and 0.168, respectively). BCVA improved to 0.71 ± 0.46, 0.73 ± 0.36 and 0.59 ± 0.41 LogMAR at the 6-month follow-up point (*P* = 0.039, 0.048 and 0.195, respectively). Initial MD was 19.26 ± 7.02, 20.51 ± 4.68 and 19.3 ± 7.17 in groups 1, 2 and 3, respectively (*P* = 0.6). Corresponding values at month 1 were 20.26 ± 8.52, 19.52 ± 7.08 and 18.3 ± 7.45, (*P* = 0.6); and at month 6 were 18.42 ± 8.17, 17.66 ± 6.44 and 16.53 ± 6.32, respectively (*P* = 0.6). RNFLT at presentation was 166 ± 57, 184 ± 57 and 193 ± 65 μm in groups 1, 2 and 3, respectively (*P* = 0.2), which decreased to 73 ± 11, 87 ± 26 and 79 ± 19 μm at the final follow-up (all *P* < 0.001)	Exclusion of patients with diabetes mellitus or poorly controlled hypertension.
Weiss (2017) [41]	IV	10 total	Case series	(1–35 years)	Autologous bone marrow-derived stem cell therapy	VA improvement in bilateral vision in 80% of patients (*P* = 0.02) with an average of 3.53 Snellen lines. 73.6% of eyes treated gained vision (*P* = 0.01) and 15.9% remained stable in the post-operative period. The average LogMAR change in treated eyes was a gain of 0.364 (*P* = 0.008).	N/A
Pakravan (2017) [42]	III	83 treated 30 untreated	Case–control	14 days	IV erythropoietin and steroid /steroid alone	No significant difference between the 3 groups for BCVA (*P* = 0.8), MD (*P* = 0.8), RNFLT (*P* = 0.1) at 6 months	Hypertension, dyslipidaemia, ischaemic heart disease Exclusion of patients with diabetes mellitus or poorly controlled hypertension
Saxena (2018) [22]	II	19 treated 19 untreated	Double-blind randomised	1 month	Oral steroids (80 mg tapering dose)	Untreated group showed a median baseline BCVA of 0.8 LogMAR (range, 0–2.7 LogMAR), whereas treated group showed a median baseline BCVA of 1 LogMAR (range, 0.5–3 LogMAR; *P* = 0.16). The final median BCVAs of the untreated group and treated group were 0.6 LogMAR (range, 0–2.7 LogMAR) and 0.5 LogMAR (range, 0.2–1.8 LogMAR), respectively (*P* = 0.78). Both groups showed a statistically significant improvement in BCVA from baseline during 6-month follow-up (*P* = 0.01 and *P* = 0.003 for the untreated and treated groups, respectively); however, the treated group showed a greater change in vision compared with the untreated group. Superior and inferior RNFLT showed a greater reduction of oedema at the 1-month follow-up visit (*P* = 0.028 and *P* = 0.031, respectively) in the treated group compared with the placebo group. There was a greater change in the superior and inferior quadrants (*P* = 0.03 and *P* = 0.03, respectively) at the 1-month follow-up visit in the treated group compared with the untreated group. The percentage change in RNFLT in both groups was statistically similar.	Hypertension, dyslipidaemia, obstructive sleep apnoea Exclusion of patients with diabetes
Kalabova (2020) [43]	III	55	Retrospective	N/A	IV vasodilators alone or with IV corticosteroids	In the group treated only with IV vasodilator, mean VA at the beginning of NAAION was 0.356, and immediately after the end of therapy 0.439. Thus, VA improved by an average of 0.083. In the group with combined therapy, average VA before treatment was 0.398 and immediately after the end of therapy 0.429, i.e. VA improved by an average of 0.031. No significant difference was found between the groups in the change of VA (*P* = 0.7).	Hypertension, diabetes mellitus, dyslipidaemia, hypercoagulation states (hyperhomocysteinaemia), collagenosis, nocturnal hypotension, obstructive sleep apnoea syndrome, treatment for erectile dysfunction and smoking
Nikkhah (2020) [44]	II	Group A (systemic erythropoietin): 33 Group B (oral steroids): 32 Group C (control): 32	Randomised controlled	5 days	10,000 units of erythropoietin / 12 h for 3 days (group A)/ oral prednisone 75 mg /24 h tapered off in 6 weeks	55% of patients in group A (systemic erythropoietin) versus 34.3% in group B (oral steroids) and 31.2% in group C (control) had an improvement of at least 3 lines in the best-corrected VA at M6 (*P* = 0.04).	Exclusion of patients with systemic conditions such as diabetes or uncontrolled high blood pressure
Durbant (2021) [26]	III	41 treated 27 untreated	Retrospective unmasked and non-randomised	N/A	Intravitreal triamcinolone (4 mg/ 0.1 ml)	Higher proportion of patients improved VA by 2 lines or more in the treated group (49%) compared with the untreated group (11%, *P* = 0.01). Among patients injected before 15 days, the proportion improving by 2 lines or more (55% vs. 11%, respectively, *P* = 0.01) and by 3 lines or more (45% vs. 11%, respectively, *P* = 0.03) were significantly higher than in the untreated group. Visual field improvement was only observed in the subgroup of patients injected within 15 days with a significant improvement of the mean deviation (dB) within 6 months (*P* = 0.01).	N/A

*BCVA* best-corrected visual acuity, *EECP* enhanced extracorporeal counterpulsation, *FC* Fluocortolone, *IV* Intravenous, *MD* mean deviation, *N/A* not applicable, *PFX* Pentoxifylline, *PO* per os, *PSD* pattern standard deviation, *RNFL* retinal nerve fibre layer, *RNFLT* retinal nerve fibre layer thickness, *VA* visual acuity, *VAR* visual acuity rating, *VF* visual field

After unilateral involvement, bilateralisation at 5 years can occur in 9.5–24% of cases [45–47], causing severe visual disability. We recently showed that non-adherence to continuous positive airway pressure treatment by patients with severe obstructive sleep apnea syndrome (OSAS) increases the risk of contralateral NAAION by 5.5 [47].

Among novel strategies aimed at treating NAAION, treatment with a systemic endothelin receptor antagonist has recently emerged as a promising new strategy, for the following reasons:

- NAAION is associated in 75–80% of cases with OSAS [47–49]. The endothelin system is activated in OSAS patients, and endothelin 1 (ET-1) is associated with intermittent hypoxia, a major feature of OSAS [50, 51]. It is also involved in the physiopathology of cardiovascular disease (hypertension, myocardial infarction and/or vascular remodelling, all frequent comorbidities of OSAS).
- Endothelin 1 is implicated in vascular damage of the optic nerve. In animal models, the application of ET-1 directly to the optic nerve, or in the eyes, induces ischaemic optic neuropathy. An increase in the plasma levels of ET-1 was also found in NAAION patients [52].
- At the ocular level, bosentan, a duel ET-1 receptor antagonist, increases retinal and optic nerve blood flow in healthy humans and glaucoma patients [53].
- Bosentan is an already certified medication used in pulmonary arterial hypertension, and for digital ulcers associated with systemic sclerodermia, and has a good tolerance profile. While abnormally high levels of hepatic enzymes occur in approximately 10% of patients, with 3% of them discontinuing treatment, any hepatic toxicity can be easily detected [54].

### Objectives {7}

The *primary objective* of this trial is to compare the outcomes after 8 weeks of oral bosentan treatment vs. placebo, evaluated by changes in the automated visual field parameters at 3 months. The 3-month timeframe was chosen to assess both functional and anatomic evolution (resolution of optic disc swelling) [55].

The *secondary objectives* are to compare the peripapillary retinal nerve fibre thickness (RNFL) at 3, 6, 12 and 24 months after inclusion, visual function, i.e. visual acuity (VA) and visual field (VF), at 6, 12 and 24 months, quality of life at 3 and 12 months, inflammation markers and plasma levels of pre-proendothelin at 3 months, as well as the extent of bilateralisation of NAAION at 24 months, between the two groups (bosentan and placebo) of patients.

### Trial design {8}

**Fig. 1:**
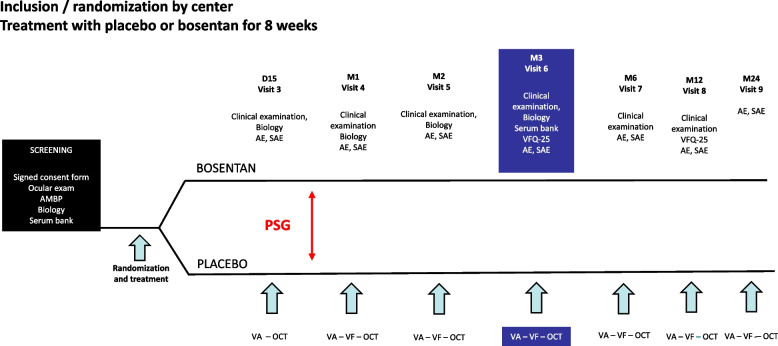
Trial design. PSG: polysomnography; AMBP: ambulatory blood pressure measurement; AE: adverse event; SAE: serious adverse event; VFQ-25: Visual Function Questionnaire 25; VA; visual acuity; VF: visual field; OCT: optical coherence tomography

This is a phase III, interventional, prospective, multicentre controlled randomised double-blind trial. The design of the trial is presented in Fig. 1.

## Methods: participants, interventions and outcomes

### Study setting {9}

This is a multicentre study including patients recruited in 6 French University (or University affiliated) Ophthalmology Centres, with recognised expertise in Neuro-Ophthalmology (Grenoble-Alpes, Bordeaux, Saint-Etienne, Angers and Paris). In Paris, two Academia-affiliated centres with high recruitment and expertise in neuro-ophthalmic disorders, including NAAION, are participating to the study (the Quinze-Vingts National Hospital Centre for Ophthalmology (CHNO) and the Adolphe de Rothschild Foundation Hospital).

### Eligibility criteria {10}

NAAION is diagnosed based on the standard criteria of recent (≤ 21 days), rapid and painless onset of VF loss, occurring with diffuse or sectorial swelling of the optic disc [56]. The trial’s inclusion and non-inclusion criteria are listed in Table 2. In each participating centre, possible differential diagnoses are carefully assessed by physicians with expertise in neuro-ophthalmology.

**Table 2:** Inclusion and non-inclusion criteria

Inclusion criteria	Non-inclusion criteria
Age ≥ 50 years old	Pregnant or breast-feeding women^a^
Patients who signed the consent form	Patients with other acute or chronic intercurrent ocular pathology interfering with visual acuity or visual field (diabetes, drug-induced or other retinopathy, other optic neuropathy including uni- or contralateral glaucoma and/or intraocular pressure > 30 mmHg, advanced cataract, corneal opacities, amblyopia < 5/10, severe myopia > −6 diopters, retinal disease)
Patients affiliated with a national health insurance scheme or beneficiaries of such a scheme.	Simultaneous bilateral NAAION, occurring 1 month apart or less
	Evidence or suspicion of other causes of optic neuropathies considered as main differentials: 1/ giant cell arteritis causing arteritic anterior ischemic optic neuropathy (AAION) 2/ Optic neuritis (based on clinical examination, personal history of multiple sclerosis, contributive ancillary investigations) and 3/ compression/infiltration of the optic nerves, based on the clinical findings and orbital/brain imaging.
	Patients with systolic blood pressure below 100 mmHg
	Patient with orthostatic hypotension (20 mmHg drop in SBP and/or 10 mmHg drop in DBP when moving to a standing position)
	Neurological history of vascular or tumour-related changes to the visual field or other optic neuropathy
	Systemic inflammatory disease
	Known allergy to bosentan
	Patients with moderate to severe hepatic impairment (Child-Pugh class B or C), biliary cirrhosis (serum levels of liver aminotransferases, aspartate aminotransferases (ASAT) and/or alanine aminotransferases (ALAT), greater than three times the upper limit of normal, bilirubin greater than twice normal)
	Estimated glomerular filtration rate (GFR) < 30 ml/min/1.73 m^2^
	Patients treated with drugs whose efficacy may be reduced by activation of cytochrome P450, 2C9, 3A4 and 2C19 isoenzymes
	Patients treated with amiodarone
	Patients treated with one of the prohibited concomitant treatments in the study
	Patient treated with systemic corticosteroids (background treatment or treatment initiated at the time of NAAION diagnosis)
	Person deprived of liberty by judicial or administrative decision, adult protected by law, hospitalised person
	Ongoing participation in another clinical research study or in the exclusion period of another clinical study

^a^Due to potential pharmaceutical interactions, bosentan may render hormonal contraception ineffective. Therefore, women using hormonal contraception as the only method of contraception should be advised to use a complementary method of contraception or to use another reliable method of contraception. If there is any doubt about which method of contraception is most appropriate for the individual patient, the advice of a gynaecologist is recommended

### Who takes informed consent? {26a}

Eligible patient’s written informed consent is obtained by a trial investigator, in the presence of a witness, after clear and detailed information has been provided to the patient and before any trial procedure is carried out. Participation is voluntary, participants can withdraw at any time, and inclusion in the trial does not affect the patient’s usual care.

### Additional consent provisions for collection and use of participant data and biological specimens {26b}

Permission for serum banking is included in the informed consent. Patients are informed that their anonymised data might in the future be the subject of clinical research and they can oppose this by informing the investigator.

## Interventions

### Explanation for the choice of comparators {6b}

The choice of placebo as a comparator was made on the basis of the absence of any efficient and available drugs for the treatment of NAAION.

### Intervention description {11a}

The treatments are initiated after inclusion and randomisation according to the group to which the patient is assigned: Group 1: oral 125 mg bosentan twice a day (BD) (total dose, 250 mg/day); Group 2: oral placebo, BD. Treatments are administered for 8 weeks.

The coordinating pharmacy prepared the investigational medicinal products (IMP), obtained from Pharmascience Inc. Montréal, Québec. Masking of pms-BOSENTAN 125 mg and 62.5 mg tablets was done by overencapsulation in size #1 opaque capsules. Pms-BOSENTAN 125 mg placebo and pms-BOSENTAN 62.5 mg placebo are empty capsules overencapsulated in the same size #1 and colour opaque capsules. The coordinating pharmacy also performed the packaging and labelling of the IMP and matching placebo. Thus, IMPs and placebos are indistinguishable.

### Criteria for discontinuing or modifying allocated interventions {11b}

No adjustments in dosage according to age are planned, in line with the Summary of Product Characteristics (SmPC). A reduction in dosage is foreseen in case of any increase in liver transaminases (Table 3). The normal bosentan dosage can be resumed only if the level of transaminases has returned to their baseline value. Transaminase levels must be verified 3 days after resumption of bosentan, and then every 2 weeks. Similarly, a reduction in the dosage of bosentan (or its interruption) is planned in case of a sustained fall in systolic blood pressure (Table 3).

**Table 3:** Algorithm for reducing dosage or discontinuation of bosentan depending on hepatic transaminases and systolic blood pressure

	Modification	Action to take
**Hepatic transaminase**		
> 3 and ≤ 5 × LSN	Reduce the daily dose by half (i.e. 62.5 mg morning and evening in this trial) and check transaminase level at least every 2 weeks.	If the level returns to its starting value, continue or resume bosentan treatment if applicable.
> 5 and ≤ 8 × LSN	Interrupt treatment and verify transaminase level at least every 2 weeks.	Once the rate has returned to starting value, resume bosentan treatment.
> 8 × LSN	Definitively interrupt bosentan treatment.	In case of high transaminase level with clinical signs of liver disorder (e.g. nausea, vomiting, fever, abdominal pain, jaundice or unusual lethargy or fatigue) or high bilirubin level equal to or greater than twice the LSN, bosentan treatment should be interrupted definitively.
**Systolic blood pressure**		
SBP < 100 mmHg		Re-verify BP at investigating centre
90< SBP < 100 mmHg		Verify BP the next day at investigating centre
Persistent 90< SBP < 100 mmHg		Self-measurement verification of BP every week until normalisation
SBP < 90 mmHg	Reduce dose (bosentan/placebo 125 mg) by half to (bosentan/placebo 62.5 mg)	Check SBP for 3 days by self-measurement = medical verification of self-measurement results (patient brings device to hospital)
		SBP < 90 mmHg = discontinue treatment
		SBP > 90 mmHg = continue half dose
SBP < 90 mmHg more than 20 mmHg lower than SBP compared to screening	Immediately reduce dose of (bosentan/placebo 125 mg) by half (bosentan/placebo 62.5 mg)	Monitor BP for 3 additional days with self-measurement after dose reduction, then medical verification of self-measurement results (patient brings device to hospital)
		If hypotension SBP < 90 mmHg is maintained: discontinue treatment
		If SBP > 90 mmHg = continue half dose

*LSN* last seen normal

Participant withdrawal may be at the request by the patient and/or due to a severe adverse event. Furthermore, the patient’s participation in the study can be discontinued if liver enzymes exceed five times the upper limit of normal. After discontinuing the treatment, patients are seen in consultation every 2 weeks until the disappearance of their abnormal liver enzyme levels. Subjects who have withdrawn or been withdrawn from the trial are not replaced. Consequently, a greater number of subjects will be included than required by the sample size calculation.

The sponsor can stop the study at any time for the following reasons: inability of the investigators to include patients in accordance with the planned timeline, absence of signed informed consent, major violations of the protocol or incomplete or erroneous data.

In case of an adverse event deemed by the investigator to be serious and that could harm their patients’ health, the investigator can stop their participation in the study with the agreement of the sponsor.

### Strategies to improve adherence to interventions {11c}

The importance of good adherence to the study treatment is explained to the patient at the screening and inclusion visits. No other direct measures are being taken to improve adherence.

### Relevant concomitant care permitted or prohibited during the trial {11d}

As far as possible, introducing, or modifying existing, concomitant treatments should be avoided throughout the trial.

The prescription of aspirin at a dosage of between 75 and 300 mg/day is tolerated if it is indicated for cardiovascular disorders in secondary prevention for patients with a history of complicated carotid or coronary atheroma, stroke, arteriopathy of the lower limbs or aneurism of the abdominal aorta. In primary prevention, aspirin will be proposed starting in the 3rd month of the protocol, after discontinuing bosentan or placebo treatment. If necessary, continuous positive airway pressure (CPAP) treatment can be initiated after the visit at 3 months.

Simvastatin treatment requires adapting the dosage to the patient’s lipid profile (cholesterol level), given that bosentan reduces the plasma concentrations of simvastatin. Prohibited treatments or those requiring close monitoring, as well as the related risks, are summarised in Table 4.

**Table 4:** Prohibited treatments or those requiring close monitoring

**Prohibited treatments**	**Risks**
Cyclosporine A	Major risk of increasing residual plasma levels of bosentan following transport protein inhibition of bosentan in hepatocytes
	Major risk of decreasing plasma concentrations of cyclosporine with reduction in its efficacy and therefore its immunosuppressive activity
Glibenclamide	Major risk of increasing risk of elevation of liver enzymes
General corticosteroid therapy	Risk of interfering with potential efficacy of bosentan
Amiodarone	Drug giving NAAION-like picture
CYP 3A4- and 2 C9 2C19-inhibitor drugs	Risk of increasing plasma bosentan concentrations
CYP 3A4-, 2C9-inductor drugs	Risk of decreasing plasma bosentan concentrations
Protease- and ritonavir-inhibitor drugs	Risk of increasing plasma bosentan concentrations
Nevirapine	Pronounced hepatotoxicity that can potentially cumulate with that of bosentan.
**Not recommended**	**Close monitoring required**
Anti-coagulants (warfarin and other antivitamin Ks)	INR (3–4 days after beginning of treatment, after each dosage modification and after discontinuation of bosentan treatment) during treatment initiation and/or dosage increase
Hormonal contraceptives (oestrogen/progestin combination and progestin-only pills)	Risk of reducing efficacy
Sildenafil intake	Discontinuation of this drug should be discussed with the patient to be included in the protocol, especially since sildenafil is suspected of being associated with NAAION onset.
Immunosuppressors (tacrolimus and sirolimus)	Bosentan lead to increase in the concentration of tacrolimus and sirolimus

*INR* international normalised ratio, *NAAION* nonarteritic anterior ischemic optic neuropathy

### Provisions for post-trial care {30}

After the end of follow-up, patients receive normal care appropriate to the progression of their condition. If there are any harms due to trial participation, compensation is included in the assurance cover take out by the sponsor.

### Outcomes {12}

The *primary endpoint* (a) is the mean deviation in VF (in dB) 3 months after inclusion. This endpoint is considered sufficient to assess functional and anatomic recovery with the disappearance of optic disc swelling by this time [57].

The *secondary endpoints* are as follows: (b) peripapillary retinal nerve fibre thickness (RNFL) and macular ganglion cell layer thickness measured using SD-OCT at 3, 6, 12 and 24 months after inclusion; (c) visual function (VA using the ETDRS scale and VF) at 6, 12 and 24 months: (d) the extent of bilateralisation of NAAION at 24 months; and (e) quality-of-life parameters assessed using the visual function questionnaire (VFQ-25) [58] at 3 and 12 months.

### Participant timeline {13}

**Fig. 2:**
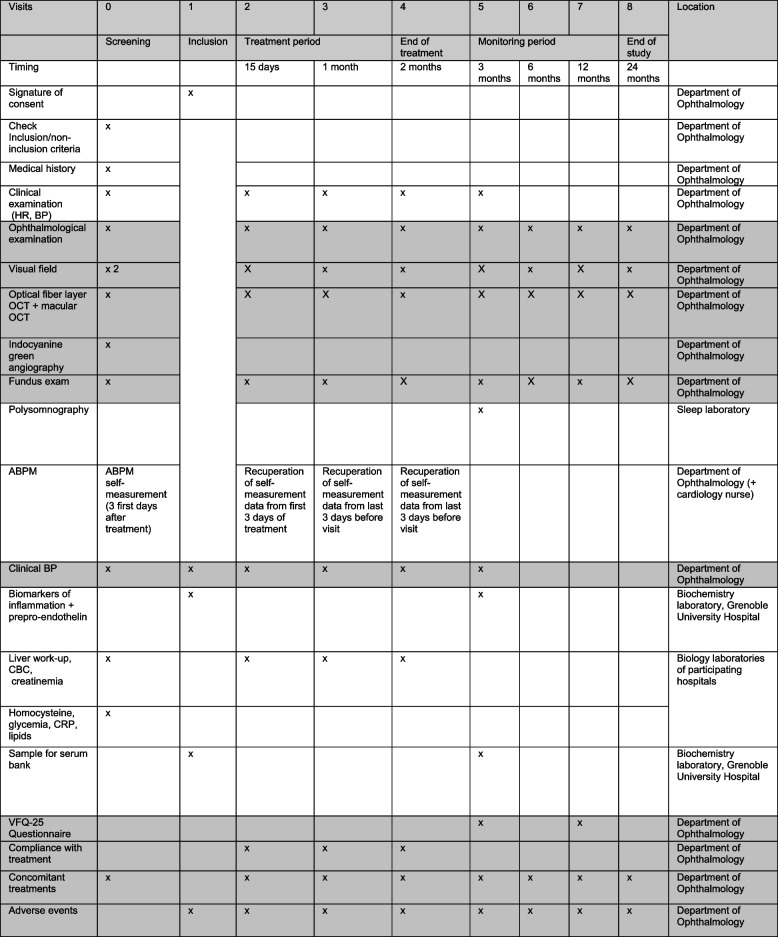
Schedule of enrolment, interventions and assessments for a patient included in the ENDOTHELION trial

The timeline for inclusion, assessments and visits for the participants are summarised in Fig. 2. Patients are seen in consultation to evaluate tolerance (including blood tests), adherence and treatment efficacy at 2 weeks, 1 month, 2 months and 3 months).

Following randomisation, the first administration of the treatment is given in the ophthalmology department. Blood pressure (BP) is checked 4 h after this, corresponding to the plasma peak of bosentan according to the pharmacological data in the study by Weber et al. [59].

Patient monitoring/follow-up includes consultations at week 2, and at 1, 2, 3, 6, 12 and 24 months with one of the investigating ophthalmologists, during which several examinations are carried out: an ophthalmological examination (VA, VF, SD-OCT and fundus examination), a clinical examination searching for the adverse side effects of bosentan, an office-measurement of BP and a blood work-up including a haemogram and measurement of liver enzymes (until and including the month 2 visit). Self-measurement of BP is programmed during weeks 1 and 4 and the last week of treatment (week 8).

The primary endpoint is set at 3 months so as to assess the efficacy of bosentan before initiating treatment for OSAS, the recognised period when visual function usually stabilises, and papillary oedema disappears, proceeding to papillary atrophy. Contreras et al. [60] showed that at 1.5 months following the acute phase, the RNFL thickness of the affected eye was similar to that of the unaffected eye, indicating that the optic disc swelling had partly resolved. After this first period, atrophy of the optic nerve head became detectable, such that at the 3-month visit, the RNFL had thinned by 39%, compared to the contralateral normal eye [60]. Loss of retinal nerve fibre layers stabilised thereafter, with only 6% additional thinning at the 12-month visit. In another study, Hayreh et al. [57] estimated the median time to papillary oedema resolution was 7.9 weeks (IQR, 5.8–11.4 weeks).

In the present trial, at 3 months, the clinical examinations and laboratory analyses (including a study of endothelial function), the ophthalmological examinations (VA, VF, SD-OCT and fundus examination) and the blood work-up (with serum banking) performed at inclusion are repeated. A polysomnography is planned after the 3-month visit.

The visits at 6 and 12 months and the end-of-protocol visit at 24 months include an ophthalmological examination (VA, VF, SD-OCT and fundus examination). The quality-of-life questionnaire (VFQ-25) [58] is completed at the 3- and 12-month visits.

### Sample size {14}

The sample size calculation was based on VF data (primary outcome) from a published study [61], and from a retrospective analysis of the patient cohort at Grenoble-Alpes University Hospital (personal data). Bellusci et al. [61] showed that inferior VF defects varied from −11.7 ± 2.7 at diagnosis to 10.3 ± 3 dB (*n* = 5) at 6 months, with diffuse defects varying from −15.5 ± 7.4 to −16.4 ± 7.9 dB (*n* = 7) and central defects varying from −5.3 ± 0.7 to 4.8 ± 1.7 dB over the same timespan (*n* = 4). Consistently, in our retrospective cohort, the progression of inferior altitudinal VF defects (*n* = 14) showed an 11% deterioration in the mean deviation (MD) between 3 and 6 months (from −15.2 ± 9 initially to −15.8 ± 7.8 dB) and 21% worsening of the MD for superior altitudinal defects (*n* = 8, from −14.9 ± 4.2 dB initially to −17.08 ± 5.7 dB).

We hypothesised that the mean deviation at inclusion would be −15 ± 9 dB and that the treatment would provide a 40% improvement in the defect, i.e. a progression from −15 to −9 dB, while no change is expected in the placebo arm. A sample size of 36 patients per group would thus provide 90% power to detect such a difference using a general linear model, with a common standard deviation of 9, an alpha risk of 0.05 and with an *r*-squared for the covariate (VF at baseline) of 0.3. To take account of patients lost to follow-up and/or those with prematurely stopping the protocol (including interruptions related to the side effects of bosentan, evaluated at 20% of the sample, *n* = 14), a total of 86 patients need to be included (*n* = 43 per group).

This sample size should provide >90% power to detect a significant difference in the thinning of retinal nerve fibres, hypothesising a smaller reduction in RNFL thickness after treatment (reduction evaluated at −43% in the placebo arm patients versus −25% in patients treated with bosentan), using an ANCOVA.

### Recruitment {15}

All eligible patients attending one of the participating ophthalmology departments are proposed participation in this trial. The recruitment period began in August 2015, is ongoing and is expected to end in December 2022. The estimated rate of recruitment is 1 or 2 patients per month. In 2016, the study was stopped for 9 months due to a supply issue of bosentan and recruitment was slow in 2020 and 2021 due to COVID. In August 2022, 72 out of 86 patients had been recruited.

## Assignment of interventions: allocation

### Sequence generation {16a}

Centralised randomisation with computer-generated random numbers, stratified by study centre, is used to randomly assign patients who meet the inclusion criteria to the bosentan or the placebo group. A MEDSHARING IWRS (Interactive Web Response System) server is used for randomisation.

### Concealment mechanism {16b}

A MEDSHARING IWRS (Interactive Web Response System) secured server is used for randomisation.

### Implementation {16c}

The study investigator enrols participants and accesses the website using a personal ID and password during the inclusion visit and is thereby informed of the intervention assigned to the participant.

## Assignment of interventions: blinding

### Who will be blinded {17a}

The investigators, patients and the hospital pharmacies are blinded to the treatment allocated. Only the coordinating pharmacy is unblinded to the treatment batches sent to the participating centres. The bosentan and placebo tablets cannot be differentiated visually.

### Procedure for unblinding if needed {17b}

In case of a serious adverse event requiring medical intervention, unblinding is planned along with a declaration to the Clinical Trials Vigilance Unit. The investigator can obtain the unblinding directly from the IWRS website, although it is recommended that the centre contacts the sponsor and the coordinating investigator before proceeding to unblinding. The reason for the unblinding must be clearly mentioned in the source documents and in the case report form (eCRF).

## Data collection and management

### Plans for assessment and collection of outcomes {18a}

All the data from the consultations and the clinical examinations, constituting the source data, are entered and stored in an electronic CRF. Statistical analysis will only be performed after verification of data entry and of the consistency of the data.

The following *ophthalmological examinations* are performed during the study:

- VA assessed using the ETDRS (Early Treatment Diabetic Retinopathy Study) scale. Fluctuation of ± 5 ETDRS letters is considered to be nonsignificant;
- Average intraocular pressure values measured three times using a Goldmann tonometer in each eye;
- Central corneal thickness is measured using pachymetry in both eyes (only at the initial screening visit);
- Axial length of both eyes is measured with non-contact biometry (only at screening);
- Fundus examination and fundus colour retina imaging is done for in each eye (45 degrees) after pupillary dilation.

The ophthalmoscopic description includes the following: the topography of the papillary oedema optic disc swelling, the retinal oedema at the posterior pole, vertical cup/disc ratio and the size of the optic disc (for the non-affected eye, the measurement takes into account the type of examination lens used) as well as the state of any posterior vitreous detachment. At the screening visit, fundus images of the nine quadrants are taken, and then after one photo centred on the optic nerve and one centred on the macula.

At inclusion the *visual field* (VF) is evaluated using automated perimetry (Humphrey 30-2 SITA standard programme) for both eyes (the healthy eye first and then the affected eye), and repeated at 24-h time intervals. Goldmann perimetry is done if automated perimetry is not feasible. The VF is classified according to the type of defect (11 possible types [62]): diffuse, superior or inferior hemifield, superior or inferior arcade, superonasal, inferonasal, superotemporal, inferotemporal quadrant, central scotoma or concentric defect.

The quantitative analysis is based on the mean deviation in decibels (dB). The minimum significant change is 2 dB between two examinations so as to take into account learning and long-term fluctuations. The VF assessment is considered reliable if the rate of fixation loss, false negatives or false positives is less than 33%.

Given its availability in all the clinical sites, we selected to use the Cirrus OCT 5000 (Carl Zeiss Meditec, Dublin, CA, USA) for *Optical Coherence Tomography* (SD-OCT) with an acquisition protocol and RNFL thickness analysis, optic disc cube 200×200, study of the ganglion cell complex (GCC) and a macular cube 200×200 protocol, all with the healthy contralateral eye examined first. The measurement is validated if three good-quality images (signal power >6) are obtained.

The individual values of average RNFL thickness, either total or by quadrant (temporal, nasal, superior, inferior), measured in micrometres will be used for statistical comparisons. The OCT measurements of the optic disc size (diameter) and vertical cup/disc ratio of the contralateral eye will be compared. Besides RNFL thickness data, macular data are also acquired in order to detect subtle maculopathies or the presence of subretinal fluid. This also makes it possible to measure the central macular thickness (CMT) and macular ganglion cell-inner plexiform layer (GCC) thickness.

*Fluorescein and indocyanine green angiography* are performed to confirm optic disc swelling and allow to detect choroidal ischemia so as to differentiate arteritic anterior ischemic optic neuropathy from NAAION. If a patient is allergic to fluorescein, the angiography can be done using indocyanine green dye only.

*Ambulatory blood pressure monitoring* (ABPM) is done using an ambulatory blood pressure monitor is fitted during visit 1 between 08.30 am and 10.30 am and then removed and read the following day, at least 24 h after its installation. The device used is SPACELABS 90207 (validated by the British Heart Association and the Association for the Advancement of Medical Instrumentation). Blood pressure is measured every 15 min during the day and every 20 min at night.

Each recording is verified according to the following quality criteria: cuff size adapted to arm circumference (according to American Heart Association recommendations), calibration of the device (not more than 5 mmHg difference between the first measurement recorded by the device and a concomitant sphygmomanometric measurement of SBP and DBP), recording duration >24 h including at least 70% usable measurements with at least two exploitable measurements per time period (both night and day). SBP values > 260 mmHg or < 70 mmHg and DBP > 150 mmHg or < 40 mmHg are automatically eliminated. In case of inadequate measurements, we suggest refitting the device. The patients are requested to complete an activity form for the 24 h concerned by the recording.

The following parameters are collected: systolic blood pressure (SBP), diastolic blood pressure (DBP), mean blood pressure (MBP) and heart rate (HR), during the day (7.00 am to 10.00 pm) and during the night (10.00 pm to 7.00 am), including the night-time drop in SBP, DBP, MBP and HR.

For the *self-measurement of blood pressure* a blood pressure monitor (electronic OMRON MIT Elite Plus M10-IT, OMRON Healthcare Co. Ltd. Kyoto, Japan) is provided to all patients for the total duration of treatment (2 months). Patients are expected to perform two series of readings per day: in the morning before breakfast (and before taking the treatment) and at bedtime. They should take three readings 1 min apart at each session over a period of 3 consecutive days, during weeks 1, 4 and 8.

*Clinical blood pressure measurements* are made at each visit up to and including the 3-month visit, office-blood pressure in a seated position is measured after 10 min of rest (mean of three measurements taken 1 min apart). Resting heart rate is measured during the second BP measurement.

For *laboratory analyses*, an 8-mL fasted blood sample is taken during visits 1 to 4 to measure ASAT (IU/L), ALAT (IU/L), creatinine (μmol/L) and complete blood count. Two additional samples (to measure the HAb1c and homocysteine levels) are taken at visit 1.

Additional 7-mL blood samples are taken during the inclusion and post-treatment visit at month 3 (visit 5). The serum and plasma aliquots are anonymised and kept at −80°C for later complementary analyses (serum bank). No genetic analyses will be realised.

### Plans to promote participant retention and complete follow-up {18b}

The importance of attending all follow-up visits until the end of the trial is explained to all potential participants at the screening visit to ensure that only those who are able to commit to the entire protocol are included. The importance of adherence to the treatment is also explained. Appointments are organised by the local clinical research assistants (CRA) and investigators, who are responsible for organisation and communication with the patients.

### Data management {19}

Data monitoring and data management are realised on all of the eCRFs. Data from visual field and SD-OCT assessments are checked by an experienced ophthalmologist.

### Confidentiality {27}

This trial conforms to the reference methodology (MR-001) of the independent French Data protection agency (CNIL) endorsed by the trial sponsor (Grenoble-Alpes University Hospital). In practice, according to French law, patient data are anonymised when the patient is included in the study. Moreover, anonymisation and data protection are requested by the IRB and the study sponsor. Only one investigator in each centre has access to the individual patient identities linked to their identification numbers, kept in a secured server. Data entered in the e-crf is also stored in a secured server with a personal password required for access, which is restricted to the investigators and the study clinical research assistant only. The eCRF is managed by Medsharing (Fontenay Sous Bois, France).

### Plans for collection, laboratory evaluation and storage of biological specimens for genetic or molecular analysis in this trial/future use {33}

Blood samples are collected at all study visits up until the visit at 3 months for biological laboratory analysis of liver enzymes, inflammation markers and plasma levels of pre-proendothelin (Fig. 2). In addition, blood samples are taken at inclusion and the M3 visit for biobanking. Serum and plasma aliquots are anonymised and kept at −80°C for later complementary analyses (serum bank). No genetic analyses are planned.

## Statistical methods

### Statistical methods for primary and secondary outcomes {20a}

Data from all patients included in this study will be included in the final analysis, following the modified intention to treat (mITT) principle. Data may be excluded from the analysis if an automated VF assessment was not obtained and/or VF parameter values are not reliable.

The number of patients presenting a major deviation to the protocol will be listed, and a sensitivity analysis on the per protocol population will be conducted. The major deviations will be reviewed by the trial’s data and safety monitoring board (DSMB).

The quantitative variables will be presented as means and standard deviations. The qualitative variables will be presented as numbers and percentages. Statistical analysis will be performed using STATA® Software Version 14.2 or higher (Stata Corporation 4905 Lakeway Drive College Station, TX 77845 USA). Data normality will be evaluated graphically and tested using the Shapiro-Wilk test. The statistical tests will be interpreted considering a two-sided *p*-value < 0.05 as significant.

For the *primary endpoint analysis*, the progression in the MD (in decibels) of the VF between baseline (inclusion visit, D1) and the 3-month visit (M3) measured using automated perimetry, i.e. delta M3 – D1, will be compared between the two groups using a linear mixed effects model, with the group as a fixed factor. The study centre will be included in the model as a random effect, and the MD at baseline will be included as a covariate in the model.

A second model of the same type will be built, adjusted on the presence of OSAS and on other baseline parameters that potentially differ between the two groups (here the *Y* variable will be the difference in RNFL: M3 – D1). The quantitative variables will be log-transformed if their distribution is not normal, so as to satisfy the conditions required to apply linear models.

For the *secondary endpoint analyses*, the same type of analysis as that used for the primary endpoint will be used for VA. Other comparisons between the two groups will be made using the Student *t* test or the Mann–Whitney *U* test (depending on the normality of the data distribution) for quantitative variables. Qualitative variables will be analysed using the chi-squared test (or the Fisher exact test depending on the theoretical number of patients).

### Interim analyses {21b}

No interim analyses are planned.

### Methods for additional analyses (e.g. subgroup analyses) {20b}

The progression of the abovementioned parameters, between 3 and 6 or 12 months, in patients with or without OSAS treatment will be compared using a mixed model. Interactions between visit, group and treatment for OSAS will be investigated. The model will also be adjusted for the centre (random effect).

### Methods in analysis to handle protocol non-adherence and any statistical methods to handle missing data {20c}

All available data will be included in the final mITT analysis, even in case of protocol non-adherence. However, missing data will not be replaced.

### Plans to give access to the full protocol, participant-level data and statistical code {31c}

After publication of the trial results, the dataset will be made available to the research community on reasonable request to the corresponding author. This will be the object of a contract with the sponsor.

## Oversight and monitoring

### Composition of the coordinating centre and trial steering committee {5d}

A list of the trial investigators is given in Table 5. The trial is coordinated by Grenoble-Alpes University Hospital. The trial’s steering committee is responsible for protocol approval and any modifications, reception of reports from the coordinating centre, supervision of the trial, monitoring of study progress and writing and submitting an article for publication. The coordinating CRA is responsible for overall data management, management of the serum bank, communicating with the teams in the other participating sites, assistance for recruitment and the newsletter.

**Table 5:** Investigators from the participating centres

**ENDOTHELION STUDY GROUP:** Prof. Christophe CHIQUET, Dr. Catherine VIGNAL, Dr Emmanuel HERON, Dr. Philippe GOHIER, Prof. Gilles THURET, Dr Marie-Bénédicte ROUGIER, Dr Laurent FLET, Jean-Louis QUESADA, Prof Matthieu ROUSTIT, Prof. Jean-Louis PEPIN	
**Coordinating investigator:** Prof. Christophe CHIQUET, Clinique Universitaire d’Ophtalmologie, CHU de Grenoble Alpes, CS10217, 38043 Grenoble Cedex 9, France, and INSERM U1300 Hypoxie et Physiopathologie	
**Grenoble Alpes University Hospital investigators:** Prof. Christophe CHIQUET (1) Prof. Jean-Louis PEPIN (2, 3) Pr Olivier ORMEZZANO (7)	
**Other participants:** Prof. Matthieu ROUSTIT (8) methodologist Prof. Bertrand TOUSSAINT (4), biologist Candice TROCME, research engineer (4) Marylaure GAVARD, pharmacist (5) Audrey LEHMANN, pharmacist (6) Jean-Louis QUESADA, statistician (8), Zineb BAIDI, Emma El MOUZDAHIR, Mayssam BOUZEID, Claire BOLLARD, clinical research assistants (1)	
1: Clinique Universitaire d’Ophtalmologie, CHU de Grenoble, CS10217, 38043 Grenoble Cedex 9, France 2: Laboratoire d’Explorations Fonctionnelles Cardio-respiratoires et Laboratoire du Sommeil, CHU de Grenoble, CS10217, 38043 Grenoble Cedex 9, France 3: INSERM U 1300 Hypoxie et Physiopathologie, Faculté de Médecine et de Pharmacie, Grenoble, France 4: Laboratoire de Biochimie des Enzymes et des Protéines (BEP), CHU de Grenoble, CS10217, 38043 Grenoble Cedex 9, France 5: DRCI, Vigilance des essais cliniques, CHU de Grenoble, CS10217, 38043 Grenoble Cedex 9 6: Pharmacy, CHU de Grenoble, 38043 Grenoble, France 7: Cardiology Clinic, CHU de Grenoble, 38043 Grenoble, France 8: Clinical Pharmacology- INSERM CIC1406, Research Division, Grenoble Alpes University Hospital, Grenoble, France, France	
**Bordeaux University Hospital investigators:** Prof. Jean-François KOROBELNIK, Dr Marie-Bénédicte ROUGIER, Dr Emilie Tournaire, Service d’Ophtalmologie Prof. Pierre PHILIP, Dr Jean-Arthur MICOULAUD FRANCHI, laboratoire du Sommeil, CHU de Bordeaux Sandrine BUISSON, clinical research associate	
**Saint-Etienne University Hospital investigators:** Prof. Philippe GAIN, Dr Claire GUILLEMOT, Gilles THURET, Dr Marie Caroline TRONE, Service d’Ophtalmologie, CHU de Saint-Etienne Dr Isabelle COURT-FORTUNE, laboratoire du Sommeil, CHU de Saint-Etienne Marie MATRAY, clinical research associate	
**Angers University Hospital investigators:** Dr Philippe GOHIER, Prof Dan MILEA clinique Universitaire d’Ophtalmologie, Prof. Frédéric GAGNADOUX, Dr Pascaline PRIOU, Pneumologie, CHU de Angers Jeanne MULLER, clinical research associate	
**Adolphe de Rothschild Foundation Hospital investigators, Paris:** Dr Catherine VIGNAL, Dr Cédric LAMIREL, service des urgences ophtalmologiques, Fondation de Rothschild, Paris, Prof. Marie-Pia d’ORTHO, Centre de Sommeil – Neurophysiologie, Service de Physiologie – Explorations Fonctionnelles, Hôpital Bichat, Paris Emmanuel AUGE, clinical research associate	
**Paris Quinze-Vingts National Hospital Center of Ophthalmology investigators:** Dr Catherine VIGNAL, service des urgences ophtalmologiques, centre XV-XX, Paris Dr Emmanuel HERON, internist Wahiba KHEMLICHE, clinical research associate	
**Sponsor:** University Hospital of Grenoble-Alpes **Representative of the sponsor authorised to sign the protocol**: Monique SORRENTINO, General Director, CHU de Grenoble Alpes CS 2017, 38043 Grenoble Cedex 9 Tel: 33 (0)4 76 76 84 56 or 55, Fax: 033 (0)4 76 76 52 21	
**Coordinating center for the study: Grenoble Alpes University Hospital (Prof C CHIQUET)**	

### Composition of the data monitoring committee, its role and reporting structure {21a}

The data and safety monitoring committee DSMB) comprises Prof. Cracowski (Pharmacology department at the Grenoble-Alpes University Hospital, Grenoble, France), Prof. Labetoulle (Department of ophthalmology, Bicêtre Hospital, Paris, France) and Prof. Bron (Department of ophthalmology, Dijon University Hospital, Dijon, France). The DSMB will be convened in the event of an unexpected serious adverse event or in case of unusual (in terms of frequency of onset) serious adverse events, at the request of the sponsor’s pharmacovigilance manager, and also at the trial’s halfway mark (17 March 2021) to assess any safety issues related to the trial. Unblinding can be requested by this board, without the investigator or the patient gaining knowledge of the treatment allocation. Meetings will be in closed session and independent of the investigators. The DSMB can also propose to the sponsor and the coordinating investigator that the study be discontinued or request that the protocol be modified if the security of the patients is deemed insufficient.

### Adverse event reporting and harms {22}

The known adverse effects of bosentan as well as their frequency are presented in Table 6.

**Table 6:** Adverse events and their frequency for bosentan

Organ class	Frequency	Adverse event
**Haematologic and lymphatic system disorders**	Frequent	Anaemia, decreased haemoglobin level
	Infrequent	Thrombocytopaenia^a^ neutropenia, leucopenia^a^
	Undetermined frequency^a^	Anaemia or decreased haemoglobin level requiring blood transfusion^a^
**Immune system disorders**	Frequent	Hypersensitivity reactions (including dermatitis, pruritis and skin rash)^b^
	Rare	Anaphylaxis and/or angioedema^a^.
**Nervous system disorders**	Very frequent	Headaches^c^
	Frequent	Syncope^a,d^
**Ocular disorders**	Undetermined frequency	Blurred vision
**Cardiac disorders**	Frequent	Palpitations^a,d^
**Vascular disorders**	Frequent	Vasomotor symptoms, hypotension^a,d^
**Respiratory, thoracic and mediastinal disorders**	Frequent	Nasal congestion^a^
**Gastro-intestinal disorders**	Frequent	Gastro-oesophageal reflux, diarrhoea
**Hepatobiliary disorders**	Very frequent	Liver enzymes abnormality
	Infrequent	High liver aminotransferases associated with hepatitis (including possible aggravation of underlying hepatitis) and/or jaundice^a^
	Rare	Liver cirrhosis, liver failure^a^
**Skin and subcutaneous tissue disorders**	Frequent	Erythema
**General disorders and abnormalities at administration site**	Very frequent	Oedema, sodium and water retention^e^

^a^Data from post-marketing authorisation of bosentan, frequencies are based on the statistical model of the clinical trial with controls versus placebo

^b^Hypersensitivity reactions have been reported in 9.9% of patients taking bosentan and 9.1% of patients on placebo

^c^Headaches have been reported in 11.5% of patients on bosentan and 9.8% of patients on placebo

^d^These types of reactions can also be caused by the underlying disease

^e^Edemas or water/sodium retention have been reported in 13.2% of patients on bosentan and 10.9% of patients on placebo

Since market authorisation, rare cases of liver cirrhosis have been reported after prolonged bosentan treatment in multi-medicated patients presenting multiple co-morbidity factors. Rare cases of liver failure have also been reported. These cases underscore the importance of monthly monitoring of liver function throughout the bosentan treatment and the need to follow the above recommendations

The sponsor evaluates the expected or unexpected aspect of the serious adverse event using the SmPC and ascertains any possible implication of the treatment. The sponsor should declare all unexpected serious adverse effects to the European pharmacovigilance database (Eudravigilance), the French health authority (ANSM), the independent ethics committee and the investigators.

### Frequency and plans for auditing trial conduct {23}

The trial could be audited by the principal funder, the French Ministry of Health.

### Plans for communicating important protocol amendments to relevant parties (e.g. trial participants, ethical committees) {25}

The coordinating CRA and coordinating principal investigator are responsible for communicating important information concerning the trial, including any protocol amendments, to all parties concerned.

### Dissemination plans {31a}

Our objective is to publish the results of the trial in a peer-reviewed scientific journal and the result will also be disseminated via international conferences and/or seminars. The results will be reported in accordance with the CONSORT recommendations for randomised trials.

## Discussion

Although NAAION is one of the leading causes of blindness in individuals over 50 years of age, there is currently no effective treatment for this condition. Since endothelin has been implicated in vascular damage to the optic nerve, the dual endothelin receptor antagonist, bosentan, might be an effective treatment in the acute phase of NAAION. Through assessments of the VF and the RNFL thickness, this randomised trial evaluates its efficacy on the effects of the disease.

At the functional level, VF abnormalities are essential clinical criteria for diagnosis, quantification of initial functional loss and follow-up of NAAION patients. The campimetric defect is typically fascicular. A VF examination is indispensable to assess NAAION because VA is inconsistently reduced, notably in the absence of macular bundle involvement. VF damage can be easily quantified with mean deviation (MD) and pattern standard deviation (PSD) parameters, and these measurements are currently used in all NAAION studies [63–65].

Bellusci et al. [61] assessed the progression of functional loss over 6 months in a small group of untreated patients. They observed that inferior defects were stable at 6 months (from −11.7 ± 2.7 dB at baseline to −10.3 ± 3 dB (*n* = 5)), whereas diffuse defects (from −15.5 ± 7.4 dB to −16.4 ± 7.9 dB (*n* = 7)) and central defects (from −5.3 ± 0.7 to −4.8 ± 1.7 dB (*n* = 4)) tended to worsen. Contreras et al. [60] reported in a prospective study of 27 patients that mean MD worsened by 2 dB or more in 29.6%, improved by 2 dB or more in 48.2% and remained stable in 22.2% of patients.

Feldon et al. [66] investigated non-randomised patients screened for the IONDT trial and demonstrated stability of MD at 6 months compared to baseline in those presenting a superior altitudinal, an inferior arcuate or a central scotoma, a significant worsening in those with superior arcuate VD defect, and a slight improvement in those with inferior altitudinal or a paracentral scotoma.

Given the variability of the initial damage, both the qualitative (type of defect) and quantitative assessments of VF are essential. Thus, this evaluation should be performed as rigorously as possible, taking patient learning into account as well as the possibility of false negatives and false positives. The major limitation of automated VF assessment is that the VF cannot be evaluated in cases with substantial macular involvement with loss of fixation. In the IONDT study, this was the case for 11 out of 128 of the control group eyes (8.6%) and 5 out of 125 treated group eyes (4%).

On an anatomical level, optic disc swelling decreases after the acute phase and disappears in 4–8 weeks [57], being replaced by optic disc pallor, a sign of neuronal tissue atrophy. Evaluating RNFL thickness using SD-OCT provides an objective analysis of anatomical involvement using an indirect measurement of the axonal and retinal ganglion cell axon loss. Studies have shown that 2 months after NAAION diagnosis, the RNFL thickness of the affected eye is similar to that of the non-affected eye but that, at 3 to 4 months, a 40% decrease in RNFL thickness can be observed compared to the healthy eye [67, 68]. In the study by Garcia-Basterra et al. (23 eyes in the NAAION group vs 43 eyes in the control group), the mean RNFL was significantly thicker than the controls in eyes with acute NAAION (203.63 ± 81.41 μm vs 88.86 ± 10.74 μm) and significantly thinner from the 3rd month follow-up visit until the end of follow-up at 12 months (59.50 ± 11.52 μm vs 88.86 ± 10.74 μm) [55].

OCT measurements are reproducible among different OCT devices. The intra-visit reproducibility of the SD-OCT (Cirrus HD-OCT, Zeiss) is 5.12 μm (95% CI: 3.87–6.37), whereas the intra-visit reproducibility is 4.86 μm (95% CI: 3.65–6.07) for the mean RNFL thickness [69]. Initially (1–15 days after functional signs begin to appear), papillary oedema is revealed by an increase in RNFL thickness. However, the limitations of an RNFL measurement are related to presence of media opacities, automatic segmentation, masked effects and the presence of artefacts. The Cirrus OCT device used in the present protocol does not allow manual modification of segmentation and poor-quality acquisitions must be excluded. OCT acquisitions also depend on good ocular fixation, which can be difficult to obtain in some patients, notably if there is significant involvement of central vision.

Finally, patients with central involvement risk presenting an analysis bias because of the difficulty in obtaining an automated VF assessment and reliable OCT images of the optic nerve.

An interesting prospective study would be to define anatomical and functional factors at baseline that could be used to predict the anatomical–functional prognosis at 3 or 6 months in treated and untreated populations. No such data are currently available in the literature.

To date, there is no validated treatment for the acute phase of NAAION. Many authors consider the pathogenesis of NAAION as multifactorial, occurring in the context of transitory hypoperfusion of the optic nerve head secondary to systemic disturbances.

The low cup/disc ratio observed in a majority of NAAION patients may make the optic disc particularly vulnerable to fluctuations in local perfusion pressure and to papillary congestion. Ischaemic swelling of the axons may lead to capillary compression in the restricted space of the optic papilla [70, 71]. Corticosteroid therapy has been tested to reduce compression of the capillaries of the optic nerve head by reducing the oedema and increasing blood flow to the optic nerve, thus improving axon function of the surviving but non-functional optic nerves [20]. *Decompression of the optic nerve sheath* [62] was proposed to treat compartment syndrome caused by optic nerve head compression due to the oedema present in NAAION, which could lead to compression of the adjacent posterior ciliary arteries and propagation of ischaemia. The same concept is the rational for radial neurotomy [25], in which two incisions are made at the nasal edge of the optic disc to surgically open the scleral canal and theoretically reduce the compartment syndrome at the optic nerve head. However, these treatments have been shown to be ineffective.

A *neuroprotective approach* is also possible. The neuroprotective effect of brimonidine has been demonstrated in several experimental studies on a variety of animal species and in humans on pathological lesions such as retinal ischaemia [72], optic nerve injury [73, 74] and ocular hypertension [74]. Brimonidine has not been tested in the context of NAAION. A number of aspects of vision seem to be influenced by dopamine levels, notably VA, colour vision and visual sensitivity [75]. Consequently, levodopa (L-dopa), a precursor of dopamine, has been tested as a NAAION treatment in humans. Levodopa crosses the blood–brain barrier and its administration increases the dopamine level in the brain and the retina. However, in a retrospective non-randomised study reported by Johnson et al. [18], no significant difference in the MD of the VF was noted at 6 months, although there was greater VA improvement in the treated group. Transcorneal electrical stimulation, which may stimulate retinal ganglion cells and axons, was applied by Fujikado et al. [28] to three NAAION patients and to five patients with traumatic optic neuropathy. Two of the three NAAION patients showed improved VA of at least 0.3 LogMAR. At 3 months, the peripheral VF had significantly improved in three eyes (one case of NAAION and two cases of traumatic optic neuropathy), remained unchanged in four eyes (one with NAAION and three with traumatic optic neuropathy) and worsened in one eye (NAAION). More recently, intravitreal injection of a small interfering ribonucleic acid designed to temporarily block caspase 2 production has been evaluated in a double-blinded, randomised, sham-controlled study (data not available at this time) [76]. To date, none of these therapies have been validated in humans with NAAION.

Alternative approaches are to *improve ocular perfusion conditions at the optic nerve* through LDL apheresis or bosentan treatment. *LDL apheresis* selectively eliminates fibrinogen, LDL, cholesterol, triglycerides and LP from plasma using extracorporeal circulation. This can reduce fibrinogen and LDL by approximately 50% after a single procedure and immediately improves haemorrhagic status [77]. In a 3-month prospective, randomised, controlled trial [78], 40 NAAION patients underwent heparin-induced extracorporeal fibrinogen or LDL precipitation (HELP) treatment, haemodilution or intravenous perfusion of pentoxifylline. While no significant difference was found between the two HELP and the haemodilution groups for VA, a 2-line or greater improvement was obtained in 47% of the HELP group, 10 (52.6%) remained stable and none worsened. In the haemodilution group, VA increased in seven patients (33%), nine (42.8%) remained stable and five (23.8%) experienced a decrease. The mean calculated VF sensitivity improved significantly (*P* < 0.01) in the HELP group, increasing from 6.83 ± 4.52 dB to 8.27 ± 4.89 dB, but was not significantly changed in the haemodilution group (6.25 ± 4.12 dB to 6.12 ± 3.92 dB). The change in mean differences of the two groups was significant (*P* < 0.005).

The *endothelin receptor antagonist* bosentan increases retinal blood flow at the optic nerve head in healthy humans and glaucoma patients [53, 79]. Endothelin is also strongly implicated in cardiovascular disorders (hypertension, infarction, vascular remodelling) and the intermittent hypoxia due to OSAS, present in 70–85% of patients with NAAION [3–6]. This suggests that bosentan is a good candidate to reduce the vasoconstricting effect of endothelin in the acute phase of NAAION and may improve optic nerve perfusion conditions.

The most significant side effect of bosentan is liver dysfunction (detected via increased transaminase levels). The physiopathology of this hepatic involvement is poorly understood, but is probably related to the effect of bosentan on biliary excretion. In the largest placebo-controlled study [80] to date, the reported incidence of an abnormal increase in plasma transaminase level (by over threefold) varied from 9.7 to 14.9%, of which 3.0% were eight times the upper limit of normal. In an observational study over a longer period, 4.2% were above eight times the upper limit of normal [81]. In the European post-marketing monitoring of bosentan, of 4623 treatment-naïve patients, 352 presented an increase in transaminases, corresponding to a raw incidence of 7.6% and an annual rate of 10.1%. In the field of pulmonary hypertension, bosentan treatment was interrupted because of a rise in aminotransferases in 150 (3.2%) patients naïve to the drug [54]. Thus, transaminase levels should be measured before initiating treatment, then monthly.

A decrease in haemoglobin level has been observed in approximately 5% of treated patients [80]. This may be related to a dilution effect caused by endothelial receptor blockage in renal glomerules.

Other side effects reported after the initiation of bosentan treatment include headache (15%), flushing and loss of consciousness, probably related to its action on the systemic vascular system and the associated vasodilatation.

In in the present trial, the hepatic and haematological side effects of the drug are easily monitored with blood work-ups and blood cell counts before and during treatment.

In conclusion, an endothelin receptor antagonist is a potential approach to improving the anatomical and functional prognosis of patients with NAAION. This randomised multicentre double-blind randomised controlled trial is an opportunity to assess (1) the effect of bosentan on the structure and function of the optic nerve in NAAION, (2) the effect of bosentan on the bilateralisation rate at 24 months, and (3) the tolerance profile of bosentan in this population.

## Trial status

Recruitment started in August 2015 and is currently ongoing. On 30 May 2022, 72 patients had been included and randomised. The last patient is expected to be included in December 2022. This manuscript reports protocol version 8.0 (30 May 2020).

## Data Availability

The statistician, the data manager, principal investigator and authors of the publication reporting the trial results will have access to the final trial dataset. After publication of the trial results, the dataset will be made available to the research community on reasonable request to the corresponding author. This will be the object of a contract with the sponsor.

## Abbreviations

ANSM: Agence nationale de sécurité médicament et des produits de santé, the French health authority
CRA: Clinical research associate
VA: Visual acuity
VF: Visual field
ETDRS: Early Treatment Diabetic Retinopathy Study
HR: Heart rate
LSN: Last seen normal
ABPM: Ambulatory blood pressure measurement
MD: Mean deviation
NAAION: Nonarteritic anterior ischemic optic neuropathy
RNFL: Retinal nerve fibre layer
OCT: Optical coherence tomography
BP: Blood pressure
SBP: Systolic blood pressure
DBP: Diastolic blood pressure
OSAS: Obstructive sleep apnoea syndrome
VFQ-25: Visual Function Questionnaire 25
SmPC: Summary of product characteristics
